# Significance of Soluble CD93 in Type 2 Diabetes as a Biomarker for Diabetic Nephropathy: Integrated Results from Human and Rodent Studies

**DOI:** 10.3390/jcm9051394

**Published:** 2020-05-08

**Authors:** Minyoung Lee, Ho Seon Park, Min Yeong Choi, Hak Zoo Kim, Sung Jin Moon, Ji Yoon Ha, ARim Choi, Young Woo Park, Jong Suk Park, Eui-Cheol Shin, Chul Woo Ahn, Shinae Kang

**Affiliations:** 1 Department of Internal Medicine, Yonsei University College of Medicine, Seoul 03722, Korea; lmycj@yuhs.ac; 2 Gangnam Severance Hospital, Yonsei University College of Medicine, Seoul 06273, Korea; PHERO2000@yuhs.ac (H.S.P.); aquamarine_v@naver.com (M.Y.C.); khzkorea@gmail.com (H.Z.K.); harabugy@naver.com (J.Y.H.); ARIM1114@yuhs.ac (A.C.); PJS00@yuhs.ac (J.S.P.); ACW@yuhs.ac (C.W.A.); 3 Severance Institute for Vascular and Metabolic Research, Yonsei University College of Medicine, Seoul 03722, Korea; 4 Department of Internal Medicine, International St. Mary’s Hospital, Catholic Kwandong University College of Medicine, Incheon 22711, Korea; moonsj75@hanmail.net; 5 Y-Biologics Inc., Daejeon 34014, Korea; ywparkb@gmail.com; 6 Graduate School of Medical Science and Engineering, Korea Advanced Institute of Science and Technology, Daejeon 34141, Korea; euicheols@kaist.ac.kr

**Keywords:** sCD93, diabetic nephropathy, biomarker, estimated glomerular filtration rate, albumin-to-creatinine ratio

## Abstract

Cluster of differentiation 93 (CD93) is a glycoprotein expressed in activated endothelial cells. The extracellular portion of CD93 can be secreted as a soluble form (sCD93) under inflammatory conditions. As diabetic nephropathy (DN) is a well-known inflammatory disease, we hypothesized that sCD93 would be a new biomarker for DN. We prospectively enrolled 97 patients with type 2 diabetes and evaluated the association between serum sCD93 and DN prevalence. The association between CD93 and development of DN was investigated using human umbilical cord endothelial cells (HUVECs) in vitro and diabetic db/db mice in vivo. Subjects with higher sCD93 levels had a lower estimated glomerular filtration rate (eGFR). The sCD93 level was an independent determinant of both the albumin-to-creatinine ratio (ACR) and the eGFR. The risk of prevalent DN was higher in the high sCD93 group (adjusted odds ratio 7.212, 95% confidence interval 1.244–41.796, *p* = 0.028). In vitro, CD93 was highly expressed in HUVECs and both CD93 expression and secretion were upregulated after lipopolysaccharides (LPS) stimulation. In vivo, peritoneal and urine sCD93 levels and the renal glomerular expression of CD93 were significantly higher in the db/db mice than in the control db/m+ mice. These results suggest the potential of sCD93 as a candidate biomarker associated with DN.

## 1. Introduction

Cluster of differentiation 93 (CD93) is a type 1 transmembrane glycoprotein composed of C-type carbohydrate-recognition domains, five epidermal growth factor (EGF)-like domains, and a single transmembrane domain [1]. CD93 is mainly expressed in monocytes and endothelial cells [2]. Although the exact role of CD93 in the body is still largely unknown and currently under investigation, it is possibly an adhesion molecule [3]. CD93 is also present as a soluble form (sCD93), which is an enzymatically cleaved ectodomain of CD93 [4,5], and the sCD93 level or CD93 polymorphisms have been shown to be associated with various inflammatory diseases, such as cerebral ischemia [6], coronary artery disease [7], systemic sclerosis [8], asthma [9], and nasopharyngeal cancer [10].

Diabetes is fundamentally an inflammatory disease that leads to chronic vascular complications, such as diabetic nephropathy (DN) [11,12]. DN is defined as increased urinary albumin excretion without any other identifiable renal diseases other than diabetes [13] and is a leading cause of end-stage renal disease. There are two clinically available markers that enable the detection of the development and progression of DN: the degree of albuminuria by using the albumin–creatinine ratio (ACR) and the estimated glomerular filtration rate (eGFR) calculated using the Chronic Kidney Disease Epidemiology Collaboration (CKD-EPI) method [14]. Because early detection of DN may enable early intervention to slow or even halt the progression of DN, it is clinically important to develop a sensitive marker for DN [13]. However, even the ACR and the eGFR may not be sensitive enough to enable the detection of all DN.

In the present study, we hypothesized that sCD93 would be a biomarker of DN incidence. Our objective was to evaluate whether sCD93 is associated with the development of DN in human subjects and to identify the possible mechanism by using a diabetic rodent model.

## 2. Materials and Methods

### 2.1. Human Study Design and Population

Subjects with type 2 diabetes (*n* = 97) were prospectively enrolled from the diabetes clinic in Gangnam Severance Hospital, Seoul, Korea, from May 2012 to April 2014. All subjects gave informed consent and the study was approved by the institutional review board of Gangnam Severance Hospital (No. 3-2012-0030). The inclusion criteria were as follows: (i) over 20 years of age, and (ii) type 2 diabetes based on the 2012 ADA guideline. The exclusion criteria were as follows: (i) concomitant malignancy; (ii) other inflammatory diseases except for diabetes (rheumatoid arthritis, lupus, etc.); (iii) steroid or nonsteroidal anti-inflammatory drug (NSAID) users; (iv) acute vascular disease (acute myocardial infarction, stroke, etc.); and (v) abnormal liver function. The prevalence of hypertension or dyslipidemia was defined as subjects with a pre-established diagnosis of hypertension or dyslipidemia or those who were taking anti-hypertensive or anti-lipidemic medication, respectively. Cerebrovascular accident (CVA) included both hemorrhagic and ischemic stroke but not a transient ischemic accident. The prevalence of diabetic retinopathy or neuropathy was identified according to the guidelines of the American Diabetes Association [15]. DN was defined as both an eGFR <60 mL/min/1.73 m^2^ and an ACR ≥300 mg/g and was classified according to the degree of the eGFR or the albuminuria.

### 2.2. Anthropometric and Biochemical Measurements

The serum was collected and stored at −80 °C. All past medical histories, including diabetic macro- and microvascular complications, were recorded as case reports by a physician. Height and body weight were measured at the time of enrolment. Blood pressure (BP) was measured in a sitting position after ≥15 min of rest. Fasting plasma glucose, total cholesterol, high-density lipoprotein (HDL)-cholesterol, and triglyceride levels were measured from peripheral venous blood samples using adequate enzymatic methods (an automated chemistry analyzer, Hitachi 7600-120, Hitachi, Tokyo, Japan) after at least 8 h of fasting. The Friedewald formula was used for calculating the low-density lipoprotein (LDL)-cholesterol levels. The ACR was calculated as the random urine albumin divided by the random urine creatinine concentration. The eGFR was calculated using the CKD-EPI equation, which involves the serum creatinine value, gender, and age [14]. To measure the serum sCD93 levels in humans, we used a previously published method for the detection of human sCD93 by sandwich enzyme-linked immune sorbent assay (ELISA) [16,17]. Considering the variance in measurement, a mean of duplicated measurements was used for analysis of serum sCD93. The CD93 human protein, a kind gift from professor Shin E. C. at KAIST, Daejeon, Korea, was used as the standard protein. MAB23791 and AF2379 from R&D Systems (Minneapolis, MN, USA) were used as capture and detector antibodies, respectively. The mouse sCD93 ELISA was performed according to the manufacturer’s recommendations (MCD930, R&D Systems, Minneapolis, MN, USA).

### 2.3. Cell Culture and Animal Experiment

The human epithelial cell line (293A), human monocyte cell line (THP-1), and human umbilical cord vein endothelial cells (HUVECs) were purchased from ATCC. The 293A was cultured in DMEM medium (11995065-065, Gibco^TM^, Life Technologies Corporation, Grand Island, NY, USA) supplemented with 10% fetal bovine serum (FBS) and antibiotics (100 U/mL penicillin and 100 μg/mL streptomycin). THP-1 was cultured in RPMI-1640 medium (11875-093, Gibco^TM^) supplemented with 10% FBS, 0.05 mM 2-mercaptoethanol and antibiotics, and HUVECs were cultured in EGM^TM^-2 medium (CC-3202, LONZA, Walkersvillie, MD, USA). The 293A and THP-1 were used for comparative analysis of CD93 expression based on the cell type. At passages 3–5, HUVECs were stimulated with 500 ng/mL lipopolysaccharides (LPS, L3024, Sigma Aldrich, Saint Louis, MO, USA) for the indicated time duration (8, 24, and 48 h), and then harvested. The CD93 expression pattern was analyzed using quantitative reverse transcription-polymerase chain reaction (qRT-PCR), immunoblotting, ELISA, and immunostaining. qRT-PCR was performed as previously described [18]. Total RNA was extracted from each cell using an RNA isolation kit (12204-01, Thermo Fisher Scientific, Vilnius, Lithuania) and reverse transcribed using a cDNA synthesis kit (18080-051, SuperScript™ III First-Strand Synthesis System, Thermo Fisher Scientific, Carlsbad, CA, USA). The amount of RNA was analyzed with the designated Taqman primer and probes (TaqMan Assay ID: hCD93; Hs00362607_m1, hGAPDH;Hs02786624_g1, Applied Biosystems^TM^ Foster City, CA, USA). The level of CD93 transcription was presented as the relative value after normalization to GAPDH. All cell experiments were repeated four times. For the animal experiments, db/m+ and db/db mice were purchased (SLC, Shizuoka, Japan) and used at specific ages. The mice were allowed access to water ad libitum and housed in a pathogen-free facility at the Gangnam Severance Biomedical Center. Whole blood was collected from the tail veins, and the serum fraction was centrifuged and stored at −80 °C in a freezer for further processing. Albuminuria and urinary sCD93 levels were measured using ELISA kits (1011, Exocell, Philadelphia, PA, USA for mouse albumin, and MCD930, R&D Systems for mouse sCD93) from the urine collected for 24 h. To analyze the peritoneal cavity fluid, the peritoneum of each mouse was washed by injecting 2 mL PBS into the peritoneal cavity. After undulation for 10 min, peritoneal fluid was recollected as much as possible [19]. After centrifugation, only the fluid fraction was used to measure the sCD93 level. For the immunoblot assay of CD93 in the glomerular and tubular portion of the kidney, the dissected renal cortex was filtered through sieves in the order of 180, 106, and 75 µm. The glomeruli were collected on the 75 µm sieve, and the tubular fragments were collected from the fraction that passed through the 75 µm sieve and processed for immunoblotting [20,21]. For the immunostaining assay, the entire kidney was harvested and fixed in 4% paraformaldehyde. The frozen sections were subjected to periodic acid-Schiff (PAS) staining to visualize the changes in the glomerulus. Immunostaining of the frozen sections, as described previously [18], was performed to assess the expression of CD31 and CD93 by using the adequate primary (CD31: 550274, BD Biosciences, San Diego, CA, USA, for mouse and M0823, Agilent DAKO, Denmark, for humans; CD93: AF2379 for humans, AF1696 for mice, R&D Systems, Minneapolis, MN, USA; DAPI: D1306, Thermo, Eugene, OR, USA) and secondary antibodies (Cy3-conjugated donkey anti-rat IgG or anti-mouse IgG and FITC-conjugated donkey anti-sheep IgG or anti-goat IgG: 712-165-153, 715-165-150, 713-095-003, 705-095-003, respectively, Jackson ImmunoResearch Inc., West Grove, PA, USA). Confocal images were obtained using the confocal LSM780 (Carl Zeiss MicroImaging GmbH, Jena, Germany) and analyzed using Image J.

### 2.4. Statistical Analyses

Variables with skewed distribution, such as sCD93 level, the ACR, and the eGFR, were log_10_-transformed for further analysis. Continuous variables with normal distribution were presented as the mean ± standard error (SE), and categorical variables were presented as absolute numbers (%). The difference in continuous variables was evaluated using an independent *t* test, and that in categorical variables was assessed using a χ2 test. The correlation between two continuous variables was analyzed and presented as Pearson’s correlation coefficient (*r*). Logistic regression analysis was used to evaluate the relative risk of DN in the high versus low serum sCD93 group. The SPSS statistical package (version 25.0; IBM, Armonk, NY, USA) was used for all statistical analyses. A *p* value of <0.05 was considered statistically significant.

## 3. Results

### 3.1. Baseline Characteristics of the Study Subjects

A total of 97 patients with type 2 diabetes were analyzed in this study (Table 1). The mean age of the study subjects was 56.4 years. The mean HbA1c and ACR levels were 6.9% and 125.9 mg/g, respectively. The patients were divided into two groups according to the median serum sCD93 level. There were no significant differences in age, sex, BMI, or the prevalence of cardiometabolic risk factors between the high versus low serum sCD93 group. However, the eGFR was low in the group with high sCD93 levels (*p* = 0.047). Although statistically insignificant, higher serum creatinine (*p* = 0.058) and urinary ACR (*p* = 0.081) levels were noted in the high sCD93 group.

### 3.2. High Prevalence of DN in the High Serum sCD93 Group

The prevalence of macro- and microvascular complications was investigated and compared between the high versus the low sCD93 group (Table 2), and no significant difference was noted in the prevalence of macrovascular complications. However, regarding microvascular complications, there were more subjects with chronic kidney disease (CKD) stage 2 or stages ≥3 in the high sCD93 group than in the low sCD93 group (34.0% vs. 12.2%, *p* = 0.011 for CKD stage 2; 12.7% vs. 2.0%, *p* = 0.043 for CKD stages ≥3). The prevalence of overt proteinuria was significantly higher in the high sCD93 group than in the low sCD93 group (14.6% vs. 2.0%, *p* = 0.029).

### 3.3. Significant Association between sCD93 Levels and DN Markers

**Figure 1:**
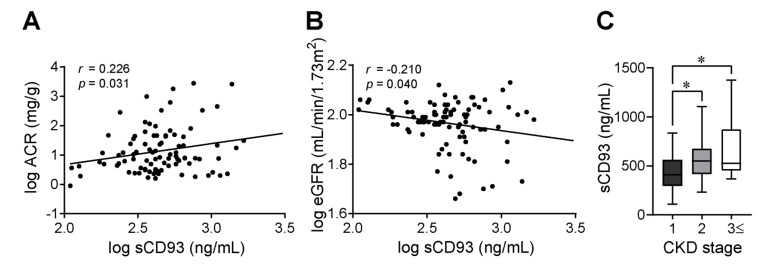
Relationship between serum sCD93 and clinical markers of diabetic nephropathy. All data were log-transformed and the correlations between serum sCD93 and (**A**) the ACR or (**B**) the eGFR were presented with Pearson’s correlation coefficient (*r*). (**C**) A graph showing the levels of sCD93 according to CKD stages.

The correlations between serum sCD93 levels and clinical parameters were evaluated (Table 3). Serum sCD93 levels showed a positive correlation with the ACR (*r* = 0.229, *p* = 0.028), and a tendency towards negative correlation with the eGFR *(r* = −0.185, *p* = 0.071). Serum sCD93 level was also significantly correlated with the log-transformed ACR and eGFR (Figure 1A,B). When compared with the serum sCD93 levels of CKD stage 1, those of CKD stage 2 and ≥3 were significantly higher and showed a gradual increase with the stage (Figure 1C). Multiple linear regression analyses were performed to verify whether serum sCD93 is an independent determinant of the eGFR and the ACR (Table 4). Demographic parameters, such as age, sex, history of hypertension, and lipid-lowering medications; glucometabolic parameters, including BMI and HbA1c; and log-transformed serum sCD93 levels were used as independent factors. Even after adjustment for these confounding factors, the serum sCD93 level was a significant independent factor for a decreased eGFR (β = −14.734, standard error (SE) = 5.564, *p* = 0.010) and an increased ACR (β = 387.943, SE = 191.129, *p* = 0.046).

### 3.4. Higher Prevalence of Renal Complications in Patients with High Serum sCD93 Levels

To determine whether elevated serum sCD93 levels were independently associated with the increased risk for DN, we evaluated the covariate-adjusted risk for either a decreased eGFR (<60 mL/min/1.73 m^2^) or macroalbuminuria (ACR ≥ 300 mg/g) and also, DN, a composite of both, in the high CD93 group compared with the low sCD93 group (Table 5). After adjusting for the covariates, the subjects in the high sCD93 group were 8.3-fold more likely to have a decreased eGFR (adjusted odds ratio [OR] 8.345, 95% confidence interval [CI] 0.846–82.353, *p* = 0.069) and 9.1-fold more likely to have macroalbuminuria (adjusted OR 9.109, 95% CI 0.990–83.837, *p* = 0.051). Furthermore, the high sCD93 group was 7.2-fold at increased risk for DN (adjusted OR 7.212, 95% CI 1.244–41.796, *p* = 0.028).

### 3.5. Enhanced Production of sCD93 from Vascular Endothelial Cells by Inflammation

**Figure 2:**
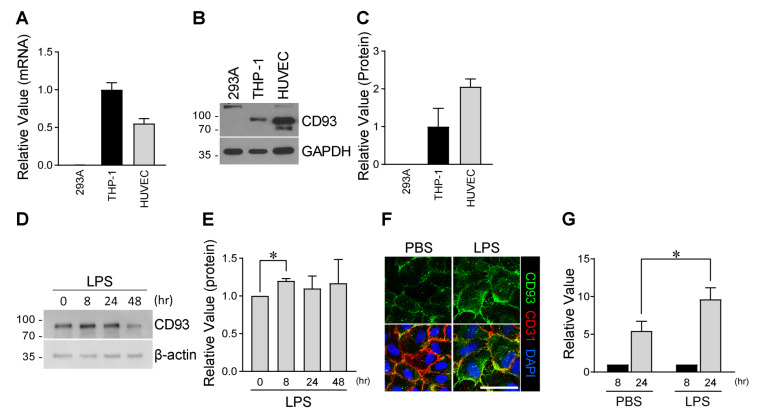
Expression of CD93 in human cells and its response to LPS. (**A**) The level of CD93 transcription was evaluated by qRT-PCR and presented as the relative value after normalization to GAPDH. The transcription level of sCD93 in THP-1 cells was set as 1 and compared between different types of cells (n = 4 per group). (**B**) The level of CD93 translation was immunoblotted with GAPDH as a loading control and (**C**) the value of CD93 relative to GAPDH was presented with the value of THP-1 as 1 (n = 4 per group). (**D**) HUVECs were harvested at the designated time point after LPS treatment and immunoblotted for CD93 expression. β-actin was used as a loading control. (**E**) The quantification of (**D**) was presented with the expression level at baseline set as 1 (n = 4 per group). (**F**) The localization of CD93 in the HUVECs was visualized by immunofluorescent staining with CD93, CD31, and DAPI after 48 h of 500 ng/mL LPS stimulation. Scale bar, 50 µm. (**G**) The supernatant of cultured HUVECs was harvested after 8 h or 24 h of 500 ng/mL LPS stimulation and the sCD93 level was measured by ELISA. All values are fold change compared to the value of 8 h without LPS stimulation as the control. The difference was compared between the PBS and the LPS group (n = 4 per group). Data are presented as the mean ± SE. * *p* < 0.05.

Because serum sCD93 was associated with the prevalence of DN in our human data, we further evaluated the underlying mechanism using an in vitro cellular and in vivo diabetic animal model. First, the expression pattern and the regulation of CD93 in immune and endothelial cells were assessed. Both cell types are active players in the inflammatory responses of diabetes [22,23]. The sCD93 mRNA levels significantly increased in both THP-1 cells and HUVECs but were barely detected in the 293A cells (Figure 2A). At the protein level, HUVECs even expressed a higher amount of CD93 than in THP-1 cells (Figure 2B,C). This indicates that not only the immune cells but also the endothelial cells can be significant sources of sCD93, the secreted soluble form of CD93. To investigate whether sCD93 can be produced from endothelial cells, HUVECs were treated with LPS and the change in CD93 expression and the amount of sCD93 in the culture supernatant were evaluated. Both CD93 expression (Figure 2D–F) and sCD93 secretion (Figure 2G) were enhanced by LPS stimulation, indicating that endothelial cells might be a major source of sCD93 in inflammatory conditions.

### 3.6. High Peritoneal sCD93 Levels and Enhanced CD93 Expression in the Glomerulus of Diabetic Mice

**Figure 3:**
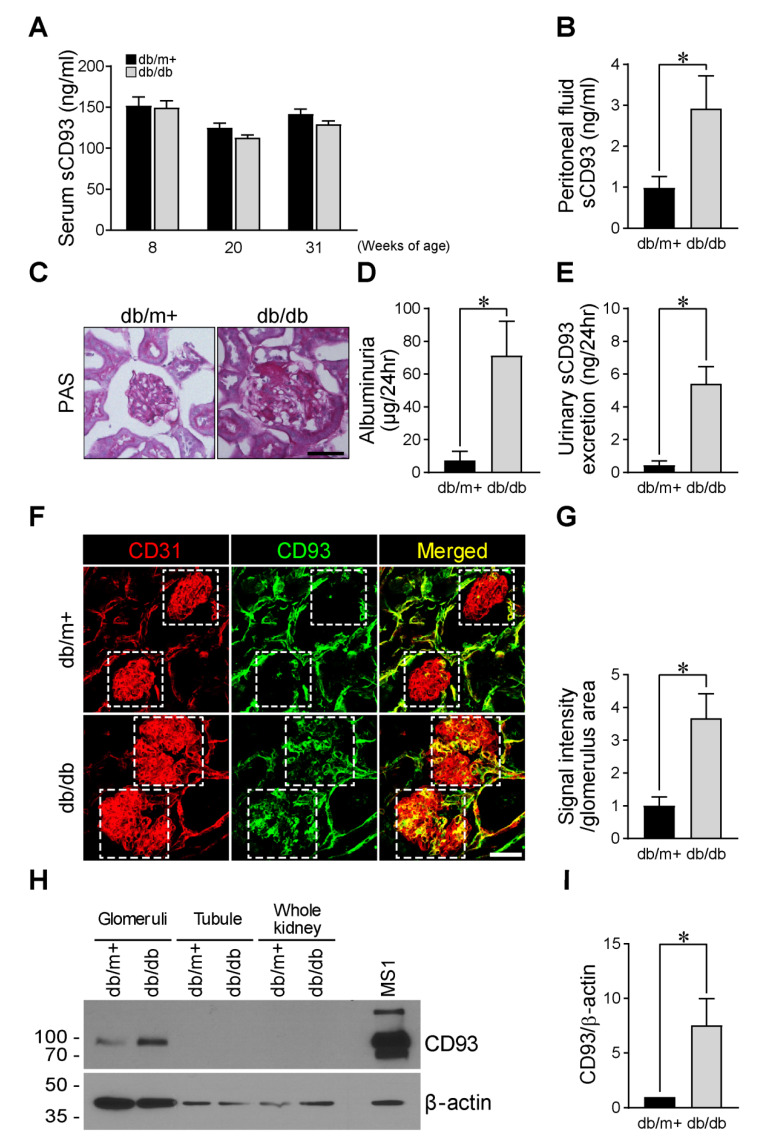
sCD93 in peritoneal lavage fluid and urine, and its expression in the glomeruli of the db/db mice. (**A**,**B**) The level of serum sCD93 at age 8, 20, and 31 weeks of age (n = 4 per group) (**A**) and the sCD93 of the peritoneal lavage fluid (n = 5~7 per group) (**B**) were measured by ELISA. (**C**–**G**) The kidney and urine samples of 31-week-old db/db mice were analyzed for the CD93 expression and sCD93 level. PAS staining (**C**), the degree of albuminuria (n = 4~5 per group) (**D**), urinary sCD93 level (n = 4~5 per group) (**E**), immunofluorescence staining with CD31 and CD93 (**F**) and the quantification (n = 5 per group) (**G**) were presented. The glomeruli and tubule were isolated from the kidney of db/db and db/m mice and processed for CD93 immunoblot with β-actin as loading control (**H**) and the quantification was presented (n = 4 per group) (**I**). The difference was compared between the db/db and db/m+ group. Data are presented as the mean ± SE. * *p* < 0.05. Scale bars, 50 µm.

The serum sCD93 concentration was not significantly different between the db/m+ and the db/db mice at 8, 20, and 31 weeks of age (Figure 3A). However, when we evaluated sCD93 level in the peritoneal cavity, another candidate site for systemic inflammatory response [24], at 31 weeks of age, the sCD93 levels in the peritoneal lavage fluid were found to be significantly higher in the db/db mice than in the db/m+ mice (Figure 3B). To address the underlying mechanism of the association between sCD93 and the ACR shown in our human data, the db/db mice with higher peritoneal sCD93 were evaluated for any difference in the degree of albuminuria. The 31-week-old db/db mice showed mesangial matrix expansion upon PAS staining (Figure 3C) and a 9.7-fold higher degree of albuminuria than that in the age-matched db/m+ mice (Figure 3D). Furthermore, urinary sCD93 levels were elevated 12-fold in the db/db mice compared with the age-matched db/m+ mice (0.45 ± 0.24 ng/24 h vs. 5.42 ± 1.02 ng/24 h, *p* = 0.014, Figure 3E). When the kidneys were immunostained for CD93 expression together with the vessel marker CD31, the CD93 expression was found to be markedly upregulated in the glomerular area in the kidneys of the db/db mice compared to the db/m+ mice, without any difference in expression in the tubule area. This suggests that there could be enhanced shedding of sCD93 from the glomerular endothelial cells together with the increased expression of CD93 in the db/db mice (Figure 3F,G). To confirm this result more specifically, the kidneys were harvested, and the glomeruli and the tubules were isolated to determine which part of the kidney would be responsible for the enhanced CD93 expression. CD93 immunoblotting suggested that the glomeruli of the db/db mice predominantly contributes to the enhanced CD93 expression (Figure 3H,I).

## 4. Discussion

In the current study, we demonstrated that serum sCD93 levels can be a novel biomarker for DN. In human subjects, the group with high serum sCD93 had a low eGFR and an increased risk of DN. Serum sCD93 positively correlated with the ACR and was an independent determinant of the ACR and the eGFR. In addition, we showed that sCD93 can be generated by LPS stimulation in human endothelial cells and that peritoneal sCD93 levels are high in diabetic mice. More importantly, CD93 expression was predominantly higher in the glomerular area of db/db mice with DN than in the control mice. These findings altogether suggest that sCD93 could be a useful biomarker for the detection of DN.

The cleavage of the extracellular portion of CD93 and the consequent generation of sCD93 are expected to occur from not only renal glomerular endothelial cells but also from other types of endothelial/myeloid cells [2,4,5,25]. However, we observed a higher CD93 expression in the glomerular capillary in the kidneys of the db/db mice than the db/m+ mice, without any difference in the tubular vasculature. These results suggest that the increased expression of glomerular CD93 may be one of the major sources for the increased sCD93 in DN.

Specific genetic loci that affect the microvascular endothelial cells in glomeruli might contribute to this phenomenon [22,26,27,28,29,30]. Although the association between sCD93 and cardiovascular disease has been studied, the results are complex, depending on the study population and the target vessels [6,7,17,31]. In our diabetic patients, there was no difference in macrovascular complications, such as coronary artery disease, peripheral artery disease, and CVA, between the low and high serum sCD93 groups. However, the incidence of DN was statistically higher and that of retinopathy and neuropathy was numerically higher in the high sCD93 group. The distinctive pathophysiology between macro- and microvascular complications [22,26,27] might contribute to this difference. Interestingly, there was no difference in the current glycemic parameters and lipid profiles between the two groups of patients with diabetes. This suggests that the production of sCD93 in patients with diabetes is not directly influenced by the current glucometabolic factors but more so by the inflammatory conditions in the diabetic milieu [22,26,27,32,33]. Considering the subgroup that shows early development and rapid progression of DN even under similar glycemic control, blood pressure, and lipid control status [26,27], identifying the unique population for high sCD93 and evaluating the mechanism of CD93/sCD93 modulation would provide further clinical implications for DN prevention and treatment.

In this study, we demonstrated the enhanced sCD93 production by inflammatory stimuli in primary human endothelial cells in vitro. Additionally, we also firstly validated the increased sCD93 production in the peritoneal cavity and urine, and higher CD93 expression in the glomeruli of diabetic mice model in vivo. We noted no difference in the serum sCD93 levels between the age-matched db/db and db/m+ mice, possibly because of the very early stage of DN that developed in the db/db mice [34,35]. It is also important to consider that mice rarely develop diabetic microvascular complications, even after long-term exposure to the diabetic milieu, in contrast to humans [34], and that the peritoneal cavity can be a prior defense location for various inflammatory stimuli [36] compared with blood circulation in mice.

Because the average life expectancy of humans is increasing, a growing number of patients with diabetes suffer from renal dysfunction in their lifetime. Although the eGFR and the ACR are used as parameters for the assessment of DN development/progression, these parameters have inherent limitations. Serum sCD93 levels can be easily measured, which may enable its use as a practical biomarker. For example, the level of sCD93 may guide physicians in deciding the diagnosis/progress/treatment plan for DN, allowing them to begin or to increase the dosage of renal-protective drugs based on an informed decision. However, there still are several points to be addressed before suggesting sCD93 as a biomarker for DN. First, it is not clear in which patient subgroup the sCD93 level is more likely to be elevated and what the elevation of sCD93 exactly means. Second, the increased sCD93 production might just be a consequence of DN owing to inflammation in diabetes or it may have an active pathophysiologic role in the progression of DN, thus providing a therapeutic target for preventing DN progression. To validate sCD93 as a practical biomarker for DN, further studies are required to determine why it is highly expressed in patients with diabetes and DN. In addition, circulating levels of several inflammatory mediators such as C-reactive protein, interleukin-6, and beta-2-microglobulin are closely associated with DN [37,38,39], and to investigate the association between these previous pro-inflammatory markers and sCD93 levels would give additional information on how to interpret the clinical implication of sCD93. Third, the level of sCD93 was mostly measured using plasma or serum in humans [4,7,9,31], and not measured using urine samples in our human study. As urinary sCD93 levels were significantly increased in the db/db mice compared with db/m+ mice in vivo, further studies using human urine samples to measure sCD93 levels could confirm the clinical utility of urinary sCD93 levels as a biomarker of DN.

The present study also has some limitations. First, the number of human study subjects was relatively small to perform additional subgroup analyses. Previously, several studies measured sCD93 in a larger number of human subjects compared with the current study [7,17,31]. However, no previous report investigated the clinical implication of sCD93 in a DN-centric view based on the eGFR and the ACR, which is the novel part of our study despite the relatively small number of participants. Second, the role of sCD93 was investigated only in diabetic kidneys, but it may also have roles in other chronic kidney disease models. Third, we defined DN using the eGFR and the ACR, but these criteria also have their own limitations for the entire DN detection. Lastly, we only analyzed the association between serum sCD93 and DN in a cross-sectional design. Thus, it remains inconclusive whether sCD93 would predict the progression of DN and a future study with longitudinally collected data is warranted.

## 5. Conclusions

To our knowledge, this is the first study that investigated the association of sCD93 with DN in human subjects. The pathophysiological role of sCD93 in DN development should be studied further, and the potential of sCD93 as an effective biomarker of DN also needs to be validated in the future.

## Figures and Tables

**Table 1:** Clinical characteristics of the study population according to serum sCD93 levels.

	Total (N = 97)	Low Serum sCD93 (*n* = 49)	High Serum sCD93 (*n* = 48)	*p*
sCD93 (ng/mL)	520.41 ± 311.34	310.71 ± 95.14	734.48 ± 309.99	**<0.001**
log(sCD93) (ng/mL)	2.65 ± 0.24	2.47 ± 0.16	2.84 ± 0.16	**<0.001**
Age (year)	56.39 ± 11.09	56.41 ± 10.48	56.38 ± 11.78	0.988
Male (***n***, %)	75 (77.3)	41 (83.7)	34 (70.8)	0.131
Hypertension (***n***, %)	57 (58.8)	29 (59.2)	28 (58.3)	>0.999
Dyslipidemia (***n***, (%))	88 (90.7)	44 (89.8)	44 (91.7)	>0.999
Duration of diabetes (year)	7.40 ± 8.39	6.80 ± 6.90	8.02 ± 9.71	0.475
DM medications (***n***, %)				
SU	24 (24.7)	12 (24.5)	12 (25.0)	0.954
Metformin	82 (84.5)	44 (89.8)	38 (79.2)	0.148
DPP4 inhibitor	53 (54.6)	28 (57.1)	25 (52.1)	0.617
Insulin	10 (10.3)	3 (6.1)	7 (14.6)	0.199
Other medications (***n***, %)				
ACE-I or ARB	50 (51.5)	26 (53.1)	24 (50.0)	0.763
Statin	56 (57.7)	27 (55.1)	29 (60.4)	0.596
Fibrate	8 (8.2)	6 (12.2)	2 (4.2)	0.268
Omega3	1 (1.0)	1 (2.0)	0 (0.0)	>0.999
BMI (Kg/m²)	25.34 ± 3.20	25.77 ± 3.41	24.89 ± 2.95	0.174
Fasting glucose (mg/dL)	133.45 ± 29.77	136.06 ± 30.91	130.72 ± 28.62	0.382
HbA1c (%)	6.89 ± 0.82	6.94 ± 0.85	6.84 ± 0.79	0.545
BUN (mg/dL)	15.29 ± 5.08	14.98 ± 3.67	15.61 ± 6.25	0.548
Creatinine (mg/dL)	0.83 ± 0.21	0.80 ± 0.15	0.88 ± 0.25	0.058
Protein (g/dL)	7.74 ± 6.74	7.07 ± 0.34	8.45 ± 9.63	0.322
Albumin (g/dL)	4.53 ± 0.29	4.54 ± 0.27	4.52 ± 0.31	0.716
ALT (IU/L)	29.05 ± 22.89	29.06 ± 14.84	29.04 ± 29.19	0.997
Total cholesterol (mg/dL)	167.17 ± 33.43	167.98 ± 28.71	166.32 ± 38.03	0.809
Triglyceride (mg/dL)	159.80 ± 103.71	165.58 ± 108.39	153.76 ± 99.44	0.583
HDL-cholesterol (mg/dL)	43.59 ± 8.98	42.59 ± 8.75	44.65 ± 9.20	0.266
LDL-cholesterol (mg/dL)	91.33 ± 30.95	90.87 ± 30.59	91.80 ± 31.66	0.887
ACR (mg/g)	125.94 ± 441.96	45.17 ± 146.79	210.30 ± 605.97	0.081
eGFR (CKD-EPI) (mL/min/1.73 m^2^)	94.26 ± 18.25	97.92 ± 12.82	90.43 ± 22.07	**0.047**

Data are described as the mean ± standard deviation (SD) or as numbers (%). The *p* values represent differences between groups determined by the paired *t* test for continuous variables and the χ2 test or Fisher’s exact test for categorical variables. Statistically significant values are indicated in **bold** (*p* < 0.05). SU, sulfonylurea; DPP4 inhibitor, dipeptidyl peptidase-4 inhibitor; ACE-I, angiotensin-converting enzyme inhibitor; ARB, angiotensin II receptor blocker; BMI, body mass index; HbA1c, glycated hemoglobin A1c; BUN, blood urea nitrogen; ALT, alanine aminotransferase; HDL-C, high-density lipoprotein-cholesterol; LDL-C, low-density lipoprotein-cholesterol; ACR, albumin-to-creatinine ratio; eGFR, estimated glomerular filtration rate.

**Table 2:** Prevalence of macro- and microvascular diabetic complications according to serum sCD93 levels.

	Total (*n* = 97)	Low Serum sCD93 (*n* = 49)	High Serum sCD93 (*n* = 48)	*p*
Macrovascular complications (***n***, (%))				
Cerebrovascular accident	4 (4.1)	2 (4.1)	2 (4.2)	0.983
Coronary artery disease	22 (22.7)	11 (22.4)	11 (22.9)	0.956
Peripheral arterial disease	3 (3.1)	1 (2.0)	2 (4.2)	0.545
No. of macrovascular complications ≥ 2	4 (4.1)	2 (4.1)	2 (4.2)	0.983
Microvascular complications (***n***, (%))				
Diabetic retinopathy	19 (19.6)	8 (16.3)	11 (23.4)	0.384
Diabetic neuropathy	9 (9.3)	4 (8.2)	5 (10.4)	0.702
Diabetic nephropathy				
CKD stage				
CKD, stage 2 (60 ≤ GFR < 90)	22 (22.9)	6 (12.2)	16 (34.0)	**0.011**
CKD, stage ≥ 3 (GFR < 60)	7 (7.3)	1 (2.0)	6 (12.7)	**0.043**
Proteinuria				
Microalbuminuria (30 ≤ ACR < 300)	17 (18.5)	10 (21.3)	7 (15.6)	0.480
Macroalbuminuria (ACR ≥ 300)	8 (8.2)	1 (2.0)	7 (14.6)	**0.029**
No. of microvascular complications ≥ 2	17 (17.5)	6 (12.2)	11 (22.9)	0.167

Data are described as numbers (%). The *p* values represent differences between groups determined by the χ2 or Fisher’s exact test. Statistically significant values are indicated in **bold** (*p* < 0.05). CKD, chronic kidney disease.

**Table 3:** Correlations between serum sCD93 and various clinical parameters.

	log(Serum sCD93)	
	*r*	*p*
Age (year)	−0.032	0.753
Duration of diabetes (year)	0.056	0.586
Body mass index (kg/m^2^)	−0.021	0.837
Fasting plasma glucose (mg/dL)	−0.104	0.312
HbA1c (%)	−0.029	0.781
Calcium (mg/dL)	0.049	0.637
Phosphate (mg/dL)	−0.052	0.618
Uric acid (mg/dL)	0.041	0.692
BUN (mg/dL)	0.026	0.804
Creatinine (mg/dL)	0.175	0.088
Protein (g/dL)	0.048	0.642
Albumin (g/dL)	−0.109	0.289
ALT (IU/L)	−0.083	0.421
Total cholesterol(mg/dL)	−0.001	0.991
Triglyceride (mg/dL)	−0.025	0.811
HDL-cholesterol (mg/dL)	−0.027	0.798
LDL-cholesterol (mg/dL)	0.049	0.645
ACR (mg/g)	**0.229**	**0.028**
eGFR (CKD-EPI) (mL/min/1.73 m^2^)	−0.185	0.071

Data are presented as Pearson’s correlation coefficient (*r*). Statistically significant values are indicated in **bold** (*p* < 0.05). HbA1c, glycated hemoglobin A1c; BUN, blood urea nitrogen; ALT, alanine aminotransferase; HDL-C, high-density lipoprotein-cholesterol; LDL-C, low-density lipoprotein-cholesterol; ACR, albumin-to-creatinine ratio; eGFR, estimated glomerular filtration rate; CKD-EPI, Chronic Kidney Disease Epidemiology Collaboration.

**Table 4:** Serum sCD93 levels as a determinant of the eGFR and the ACR.

	Univariate Model					Multivariate Model			
eGFR (CKD-EPI)	Regression Coefficient	SE	*p* Value	R^2^	Regression Coefficient		SE	*p* Value	Adjusted R^2^
Age (year)	−1.054	0.130	<0.001	0.413	−0.886		0.130	<0.001	0.523
Sex (reference: male)	−2.914	4.519	0.521	0.004	5.317		3.403	0.122	
Hypertension	−18.722	3.287	<0.001	0.257	−11.290		2.991	<0.001	
Lipid-lowering medications	−9.422	3.792	0.015	0.062	−3.451		2.851	0.229	
BMI (kg/m^2^)	−0.615	0.587	0.298	0.012	−0.094		0.431	0.828	
HbA1c (%)	−2.540	2.324	0.277	0.013	−1.228		1.642	0.457	
log(serum sCD93)	−14.025	7.681	0.071	0.034	−14.734		5.564	0.010	
	**Univariate model**					**Multivariate Model**			
**ACR**	**Regression Coefficient**	**SE**	***p* Value**	**R^2^**	**Regression Coefficient**		**SE**	***p* Value**	**Adjusted R^2^**
Age (year)	0.790	4.118	0.848	<0.001	−2.358		4.455	0.598	0.043
Sex (reference: male)	93.021	111.901	0.408	0.008	13.971		115.827	0.904	
Hypertension	187.124	93.374	0.048	0.043	193.456		103.357	0.065	
Lipid-lowering medications	109.227	96.597	0.261	0.014	81.495		98.334	0.410	
BMI (kg/m^2^)	−7.305	14.701	0.620	0.003	−14.443		14.694	0.328	
HbA1c (%)	−54.342	55.888	0.333	0.010	−40.855		55.888	0.467	
log(serum sCD93)	417.319	187.107	0.028	0.052	387.943		191.129	0.046	

Data are presented as the regression coefficient and standard error. *p* < 0.05 was regarded as statistically significant. SE, standard error; BMI, body mass index; HbA1c, glycated hemoglobin.

**Table 5:** Risk of high serum sCD93 levels for renal complications.

	GFR (CKD-EPI) < 60 (CKD, Stage ≥ 3)		
	Low Serum sCD93	High Serum sCD93	*p*
Model 1	1.00 (reference)	7.024 (0.812–60.766)	0.077
Model 2	1.00 (reference)	7.080 (0.786–63.746)	0.081
Model 3	1.00 (reference)	8.345 (0.846–82.353)	0.069
	**Macroalbuminuria (ACR ≥ 300)**		
	**Low Serum sCD93**	**High Serum sCD93**	***p***
Model 1	1.00 (reference)	8.474 (0.998–71.941)	0.050
Model 2	1.00 (reference)	8.571 (00.992–74.071)	0.051
Model 3	1.00 (reference)	9.109 (0.990–83.837)	0.051
	**Diabetic Nephropathy (CKD, Stage ≥ 3 or Macroalbuminuria**)		
	**Low Serum sCD93**	**High Serum sCD93**	***p***
Model 1	1.00 (reference)	5.566 (1.134–27.313)	0.034
Model 2	1.00 (reference)	5.991 (1.145–31.341)	0.034
Model 3	1.00 (reference)	7.212 (1.244–41.796)	0.028

Model 1, not adjusted; model 2, adjusted for age and sex; model 3, adjusted for age, sex, BMI, hypertension, lipid-lowering medications, and HbA1c. Data are presented as odds ratios, with the low CD93 group as a reference. *p* < 0.05 was regarded as statistically significant. CKD-EPI, Chronic Kidney Disease Epidemiology Collaboration; BMI, body mass index; HbA1c, glycated hemoglobin.

## Data Availability

All data generated or analyzed during this study are included in this published article.
